# Accessory Brachial Artery Causing Delayed Vasospasm After Angiography: A Detailed Analysis of a Case Report

**DOI:** 10.7759/cureus.86238

**Published:** 2025-06-17

**Authors:** Kazuyuki Mikami, Yoji Kuramoto, Takahiro Kuroyama, Yasushi Ueno

**Affiliations:** 1 Neurosurgery, Shinko Hospital, Kobe, JPN; 2 Neurosurgery, Hyogo Medical University, Nishinomiya, JPN

**Keywords:** accessory brachial artery, arterial vasospasm, brachioradial artery, cerebral digital substraction angiography, peripheral catheterization, peripheral circulatory disorder, trans-radial

## Abstract

Cerebral angiography remains the gold standard investigation for cerebrovascular disease. Transradial access is increasingly preferred over the transfemoral approach because it facilitates early ambulation and reduces complications at the access site. We report a woman in her 70s who developed delayed peripheral circulatory failure in the digital arteries following transradial cerebral angiography. After inserting a 4-French sheath into the conventional right radial artery, the catheter was advanced toward the subclavian artery but inadvertently entered a small accessory brachial artery with significant resistance, provoking severe vasospasm. The procedure was completed without incident after redirecting the catheter into the main brachial artery. Symptoms gradually appeared 24 hours after the procedure, and after 48 hours, the patient experienced burning pain, weakness, and pallor in her right hand. Angiography via the brachial artery demonstrated diffuse spasm of the radial artery, palmar arch, and digital arteries. Intra-arterial nitroglycerin quickly restored flow and alleviated symptoms. This case illustrates that inadvertent cannulation of an accessory brachial artery during transradial access can lead to delayed digital circulatory failure and that early intra-arterial vasodilator therapy is effective. Careful observation is necessary if such a catheter deviates into a small vessel with an anatomical anomaly, and spasm occurs.

## Introduction

Transfemoral access has traditionally been preferred for diagnostic angiography and endovascular therapy [1]. In recent years, however, transradial access has become increasingly popular initially in cardiology, enabling early ambulation and lowering puncture-site complications [2] and is now widely adopted in the neurointerventional field [3]. We describe a rare complication of transradial cerebral angiography in which the catheter entered a small accessory brachial artery [4,5], triggering severe vasospasm and producing delayed digital ischemia that required repeat angiography and intra-arterial vasodilator treatment. Vasospasm induced by catheter guidance into small vessels with minor anatomical abnormalities, which are occasionally encountered in daily practice, is demonstrated in this report.

## Case presentation

**Figure 1:**
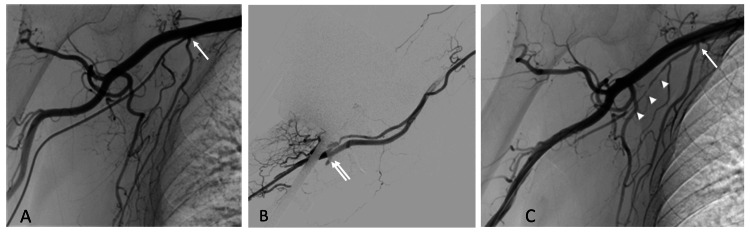
Angiographic images of the right arm at the first examination (A) Angiography through a catheter placed in the accessory brachial artery during induction. The small accessory brachial artery branched near the transition from the axillary to the brachial artery (arrow). (B) Angiography from the radial artery. The accessory brachial artery joined the main artery in the cubital region (double arrow). (C) After examination, angiography was performed through the common brachial artery. The accessory brachial artery distal to the site where the catheter was passed during guidance is spastic and delayed on imaging (arrowhead).

A woman in her 70s underwent cerebral angiography to evaluate a clipped cerebral aneurysm. A 4-French sheath was introduced smoothly into the conventional right radial artery, but the catheter met resistance as it advanced toward the subclavian artery. Road-map angiography showed that the catheter had deviated into a small accessory brachial artery running parallel to the main brachial trunk (Figure 1A). This small accessory brachial artery originated from the second part of the axillary artery close to the lateral thoracic artery and coursed parallel to the brachial artery, terminating by joining the brachial artery in the cubital fossa. After repositioning the catheter into the main brachial artery, the angiographic examination was completed uneventfully (Figure 1B). An axillary-artery injection performed before sheath removal demonstrated focal, severe vasospasm in the accessory vessel (Figure 1C); the patient remained asymptomatic.

**Figure 2:**
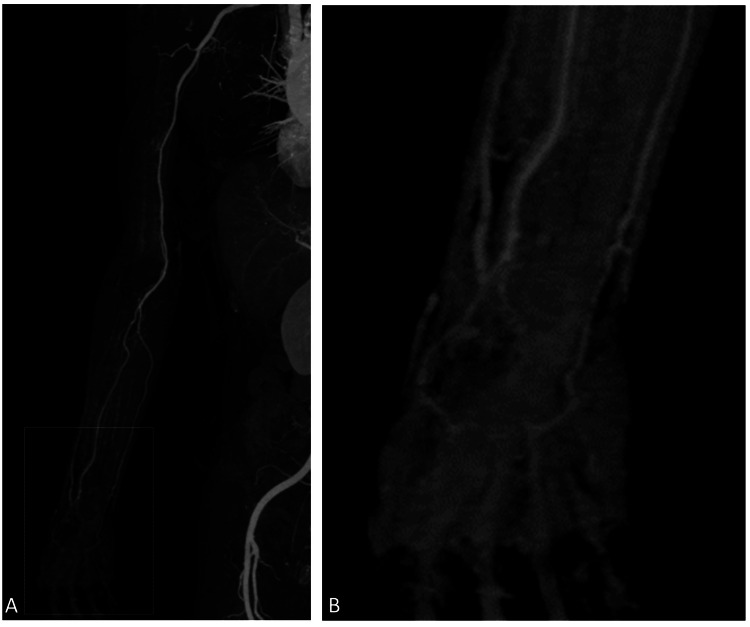
Contrast-enhanced CT at the time of emergency department (A) No occlusion of the puncture site, radial artery, or ulnar artery was patent. (B) The palmar arterial arch was partially poorly visualized but was not occluded entirely.

Hemostasis was achieved using a radial compression device (TR Band; Terumo, Japan), and the patient was discharged the following morning without any complaints. On the night of discharge, she developed burning pain, weakness, and pallor in her right hand, prompting her return to the emergency department. Radial and ulnar pulses were palpable, but intrinsic hand muscles were flaccid (manual muscle testing (MMT), grade 0/5). Contrast-enhanced CT revealed patent brachial, radial, and ulnar arteries, but there was poor opacification of the palmar arch (Figures 2A, 2B).

**Figure 3:**
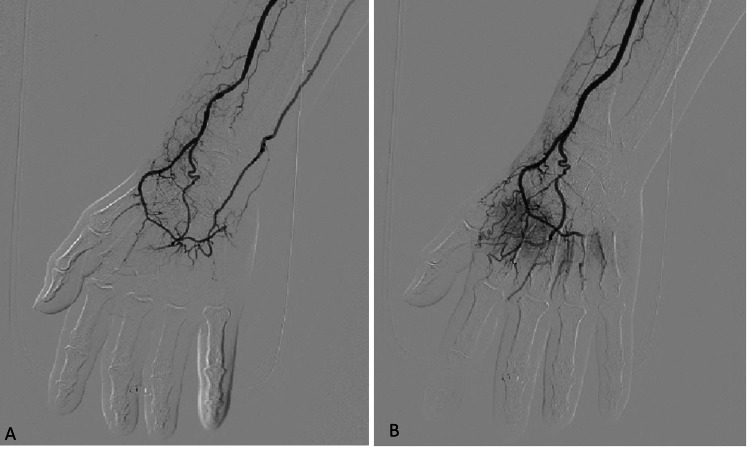
Angiographic images around the hand region at the second examination (A) Pre-treatment angiography of the radial artery. The palmar arch was diffusely spastic, and all digital arteries are obscured. (B) Post-treatment angiography of the radial artery. The vasospasm of the palmar arch improved, and the digital arteries were well-delineated.

Suspecting circulatory failure in the digital arteries, we performed additional angiography via an antegrade 4-French sheath in the right brachial artery after administering 1,000 IU of intravenous heparin. Diffuse vasospasm of the radial artery, palmar arch, and digital arteries was confirmed (Figure 3A). Intra-arterial nitroglycerin immediately restored flow and alleviated pain, resulting in complete visualization of all digital arteries (Figure 3B).

Intra-arterial nitroglycerin immediately restored flow and alleviated pain, resulting in complete visualization of all digital arteries (Figure 3B). Intravenous alprostadil (20 µg/day for three days) was also administered. Transient reperfusion edema appeared on the first day of treatment but resolved quickly after the second day. The patient was discharged on day 8, showing no symptoms.

## Discussion

Catheter angiography remains the gold standard for evaluating cerebrovascular lesions, and transradial access has largely supplanted transfemoral access because it permits early ambulation and reduces puncture‑site complications [3,6]. The commonest adverse event is radial‑artery vasospasm, mediated by α1‑adrenergic receptors and reported in 15%-30% of cases, whereas thrombosis, perforation, hematoma, pseudoaneurysm, and nerve injury together occur in roughly 10 % [7-12]. Vessel variants markedly amplify the risk of spasm [9]. Our patient harbored an accessory brachial artery, a rare (<1 %) branch that departs from the axillary or brachial artery and rejoins distally [4,5,13]. Catheter wedging inside this narrow conduit provoked severe spasm but, unusually, produced no symptoms until 24 hours later, when digital ischemia emerged. It's possible that the catheter entered a small vessel, and the initial high pressure may have led to delayed vasoconstriction. However, this is our first time facing this issue. Furthermore, establishing a clear causal relationship presents a significant challenge.

Contrast‑enhanced CT failed to reveal the lesion, underscoring its limited sensitivity for distal vasospasm. Repeat catheter angiography delineated diffuse spasm from the radial artery to the palmar arch and justified intra‑arterial nitroglycerin followed by systemic alprostadil, which rapidly restored flow and averted tissue loss. Recurrent ischemia requiring amputation has been reported after radial access [14]. Timely recognition is therefore critical. Lessons learnt from this case are summarized in Table 1.

**Table 1:** Lessons learnt from this case

No.	Lessons
1	Map aberrant anatomy immediately: Resistance during catheter advancement or an atypical trajectory warrants on‑table angiography to visualize the entire radial‑brachial axis.
2	Expect delayed ischemia: Even when a spasm initially appears benign, symptoms can surface 24–48 h later; patients must be counselled and reviewed within this window.
3	Treat hand symptoms urgently: Distal pain, coldness, or weakness after radial access mandates prompt catheter angiography, because pharmacological vasodilation is often curative.

Although we have not observed further cases of circulatory failure in the digital arteries, including procedures performed via distal radial (snuff-box) access, catheterizing small-caliber arteries poses a risk of vascular injury and vasospasm. Vigilance for variant anatomy and early intervention remain essential to prevent irreversible hand damage.

## Conclusions

Inadvertent cannulation of a small-diameter vessel with anatomical anomalies, such as an accessory brachial artery, during transradial cerebral angiography can lead to delayed circulatory failure in the digital arteries. When performing transradial angiography and interventions, we carefully identify the vessels and avoid cannulating small vessels and anatomical anomalies. Catheter angiography is superior to contrast-enhanced CT for detecting distal vasospasm, including small digital arteries, and allows for immediate intra-arterial vasodilator therapy, thereby preventing permanent hand injury.
